# Ridge planting increases the rhizosphere microbiome diversity and improves the yield of *Pinellia ternata* (Thunb.) Breit in North China

**DOI:** 10.1371/journal.pone.0304898

**Published:** 2024-09-13

**Authors:** Yaofa Li, Jingjie An, Jianglong Guo, Zhihong Dang, Zhanlin Gao

**Affiliations:** Key Laboratory of Integrated Pest Management on Crops in Northern Region of North China, Ministry of Agriculture and Rural Affairs, IPM Innovation Center of Hebei Province, International Science and Technology Joint Research Center on IPM of Hebei Province, Plant Protection Institute, Hebei Academy of Agriculture and Forestry Sciences, Baoding, China; Pan African University of Life and Earth Sciences Institute, PAULESI, NIGERIA

## Abstract

*Pinellia ternata* (Thunb.) Breit is an important traditional Chinese medicine. In North China, conventional flat planting of *P*. *ternate* is prone to root rot during the rainy season, leading to severe yield loss. Variations in planting patterns (e.g., ridge planting) can effectively alleviate this situation. However, the relationship between planting patterns and the changes induced by rhizosphere microbiome still needs to be determined. In this study, we clarified the effect of ridge planting on the yield of *P*. *ternata* and rhizosphere microbial community using high-throughput amplicon sequencing of 16S rRNA. Field experiments showed that ridge planting could increase the yield of *P*. *ternata* by 72.69% compared with flat planting. The high-throughput sequencing results demonstrated that fungal and bacterial communities in rhizosphere siols of flat and ridge planting showed obvious difference in diversity, structure, relative abundance, and community composition. The fungal phyla *Zygomycota*, *Basidiomycota*, *Glomeromycota*, and the bacterial phyla *Chlamydiae*, *Tenericutes*, and *Hydrogenedentes* were present in a higher relative abundance in the rhizosphere of ridge planting. Adonis multivariate analysis of variance results showed that 29 bacterial genera were significantly up/down-regulated, and only 4 fungal genera were changed considerably in ridge planting soil, indicating that the bacterial community composition varied significantly between the two treatments. Correlation analysis revealed that the yield of *P*. *ternata* was positively correlated with fungal genera *Emericellopsis* while negatively correlated with bacterial genera *Acetobacter*, *Iamia*, and fungal genera *Thielavia*. Overall, this study showed that ridge cropping significantly impacts the diversity and composition of the rhizosphere microbiome. It creates an environment favorable for crop growth and can be an effective planting strategy for *P*. *ternata* in areas with irrigation and high monsoon rainfall in North China.

## Introduction

Traditional medicines make vital contributions to the global healthcare industry [1], and medicinal plants have been a major source of different traditional medical systems worldwide [2]. About 30,000 medicinal plants are distributed wildly or planted worldwide, nearly one-third of which can be found in China [3]. *Pinellia ternata* (Thunb.) Breit, a member of the family Araceae, has an important position in traditional Chinese medicine [4]. It is a perennial herbaceous plant used as medicine by the bulb, with antitumor, hypolipidemic, antihypertensive, antidepressant, anti-arrhythmic, treatment of respiratory diseases and other pharmacological effects [5–7]. In recent years, with the increasing market demand and the decline in wild resources, the artificial planting area of *P*. *ternata* increased yearly [8, 9]. *P*. *ternata* is extensively distributed in most provinces of China, except for Inner Mongolia, Jilin, Heilongjiang, Xinjiang, Qinghai and Tibet. The optimum temperature for the growth of *P*. *ternata* is 15–26 °C, and the soil moisture content should be kept between 20–50% [10]. However, excessive humidity can easily induce root diseases and lead to seedling mortality [11, 12].

Conventional flat planting is still the dominant pattern of *P*. *ternate* in China [9]. However, flat planting of crops may result in severe problems during the rainy season, such as root respiration difficulties, decreased immune capacity, and serious root diseases [13]. These problems can be solved by changing planting patterns such as ridge planting, which improves the soil structure of the plow layer, increases the soil temperature and changes the rhizosphere soil moisture content [14]. In dry semi-humid areas, ridge furrows with plastic film mulching on the ridge effectively substituted for a well-irrigated planting strategy for achieving sustained agricultural development [15]. However, more than 80% of the rain in north China is concentrated in summer. Crops planting on the ridge rather than in the furrows can minimize the long-term damage from rain as much as possible. At present, ridge planting technique is gradually adopted in fruit trees, vegetables, crops, and traditional medicinal plants in North China [16–18].

Soil microorganisms are one of the important soil microenvironments and play an important role in nutrient recycling, soil biochemical processes, soil fertility, and plant growth [19, 20]. The plant rhizosphere is a major habitat for many kinds of microorganisms and is enriched with a large number of rhizobacteria that promote plant growth [21, 22]. The main role of rhizosphere microorganisms on plants is to improve their ability to obtain nutrients from the environment [23]. They also regulate plant growth and environmental adaptation through hormone synthesis or degradation [24]. Additionally, they induce plant resistance by interacting with pathogenic bacteria and initiate immune regulation [25]. However, some key factors, such as climatic conditions, soil chemical properties, and planting pattern, etc. can cause an imbalance in rhizosphere microorganisms [26, 27]. Changes in the diversity and composition of the rhizosphere microorganism would impact soil fertility, soil acidity, and the stability of soil microenvironment [27–29].

High-throughput sequencing is an important tool for analyzing microbial communities’ composition and relative abundance in complex environments [30]. Based on this technology, many studies reported that the use of rhizospheric microorganisms as biocontrol agents to regulate rhizospheric bacterial and fungal communities can help plants reduce the incidence of soil-borne disease and enhance the yield [28, 31, 32]. Previous studies have found that ridge planting alters the soil structure and physical environment [14], but its effects on rhizosphere microbial communities are unclear. So, we hypothesised that ridge planting causes changes in rhizosphere microbial communities that benefit the cultivation of *P*. *ternate*. We tested our hypothesis by investigating the influence of ridge planting on the rhizosphere microbiome using high-throughput sequencing, comparing the yield of ridge planting and flat planting during the harvest season. This study will provide a theoretical basis for ridge planting to protect seedlings and improve yields.

## Materials and methods

### Experimental design and soil sampling collection

The experiments were conducted from April to October 2020 in sandy loam soils at the Agricultural Research Farm of Hebei Plant Protection Institute, Baoding (38°57’N, 115°26’E), China. The field trial consists of two treatments: ridge planting and flat planting. In the ridge planting plot, two rows of bulbs 35 cm apart were planted on the ridge with a width of 60 cm and a height of 20 cm. Four ridges were in one replicate. Eight rows of bulbs 35 cm apart were planted flatly in one replicate. The plot experiment was repeated three times. The base fertilizer (Hubei Aotel Chemical Co., Ltd.) with 750 kg/ha was applied before sowing and irrigated two times according to local cultivation habits, on April 23^th^, May 20^th^ 2020. No pesticides were used during the experimental period. The average temperature and the rainfall from April to October 2020 in Baoding were 20.80 °C (12~26 °C) and 346.20 mm. Rhizospheric soil cores were collected from both experimental sites before harvest (October 11^th^, 2020). Roots were gently shaken, and the remaining attached tiny soils were brushed and collected in separate plastic bags as rhizosphere soil samples to analyse soil microbial community composition. Three biologically independent replicate soil samples were obtained by mixing 5 cores into one sample per treatment [26]. The samples were kept in dry ice to be transported to the lab and saved at -80 °C until the DNA extraction. Cultivation and collection were performed according to the technical regulations of *P*. *ternate* planting [33].

### Yield determination

During harvest, the number of plants in 1m rows was randomly investigated using the opposite angle line five spots method for each treatment. Tubers in 1 m rows were dug randomly, sieved to remove excess soil, rinsed with water, dried and weighed for total fresh weight. Randomized survey of 5 points per plot, 3 biological replicates per treatment.

### Soil DNA extraction and high-throughput sequencing

The total genomic DNA was extracted from 0.5 g of sample using the E.Z.N.A. ^®^ Soil DNA Kit (D5625, Omega, Inc., USA) according to the manufacturer’s instructions. Nuclease-free water was used for blank. The total DNA was eluted in 50 μL of Elution buffer and stored at -80 °C until measurement in the PCR amplification according to the standard protocols of the LC-Bio Technology Co., Ltd. (Hangzhou, Zhejiang province, China). Bacterial 16S V3-V4 region and fungal ITS genes were amplified using the 341F (5’-CCTACGGGNGGCWGCAG-3’) / 805 R (5’- GACTACHVGGGTATCTAATCC-3’) and ITS1 (5′-GAACCWGCGGARGGATCA-3’) / ITS2 (5’-GCTGCGTTCTTCATCGATGC-3’) primers set [34, 35]. PCR amplification and high-throughput sequencing were performed according to the standard protocols of the LC-Bio Technology Co., Ltd. The libraries were sequenced on an Illumina Novaseq PE250 platform.

### Bioinformatics and statistical analysis

Paired-end reads were assigned based on their unique bar code and truncated by cutting off the bar code and primer sequence. Paired-end reads were merged by the fast length adjustment of short reads (FLASH) [36]. Quality filtering on the raw reads was performed under specific filtering conditions to obtain high-quality clean tags according to the Fqtrim (v0.94). Chimeric sequences were filtered using V search software (v2.3.4). The amplicon sequence variant (ASV) table was generated with DADA2 [37] and assigned to the appropriate taxon using QIIME2’s plugin [38]. Alpha and beta diversity were calculated by randomly normalized to the same sequences. Then according to SILVA (release 132) classifier, feature abundance was normalized using the relative abundance of each sample. Alpha diversity is applied in analyzing the complexity of species diversity for a sample through 5 indices, including Chao1, Observed species, Goods coverage, Shannon, Simpson, and all the indices in samples were calculated with QIIME2. Beta diversity was calculated by QIIME2. Blast was used for sequence alignment, and the feature sequences were annotated with SILVA database for each representative sequence. Correlation analysis was performed between bacterial-fungal genera and yield according to Pearson correlation coefficient (PCC, *P* < 0.05), CorrPlot was performed using the OmicStudio tools at https://www.omicstudio.cn/tool. The relative abundance bar plots at phylum and genus level, chord diagram at the genus level, and beta-diversity heat map based on Bray-Curtis distance matrix were implemented using the R package (v3.5.2). Data were statistically analyzed using ANOVA, and means were compared using the *t*-test at *p* < 0.05.

## Results

### Effects of ridge planting on yield of *P*. *ternata*

**Fig 1:**
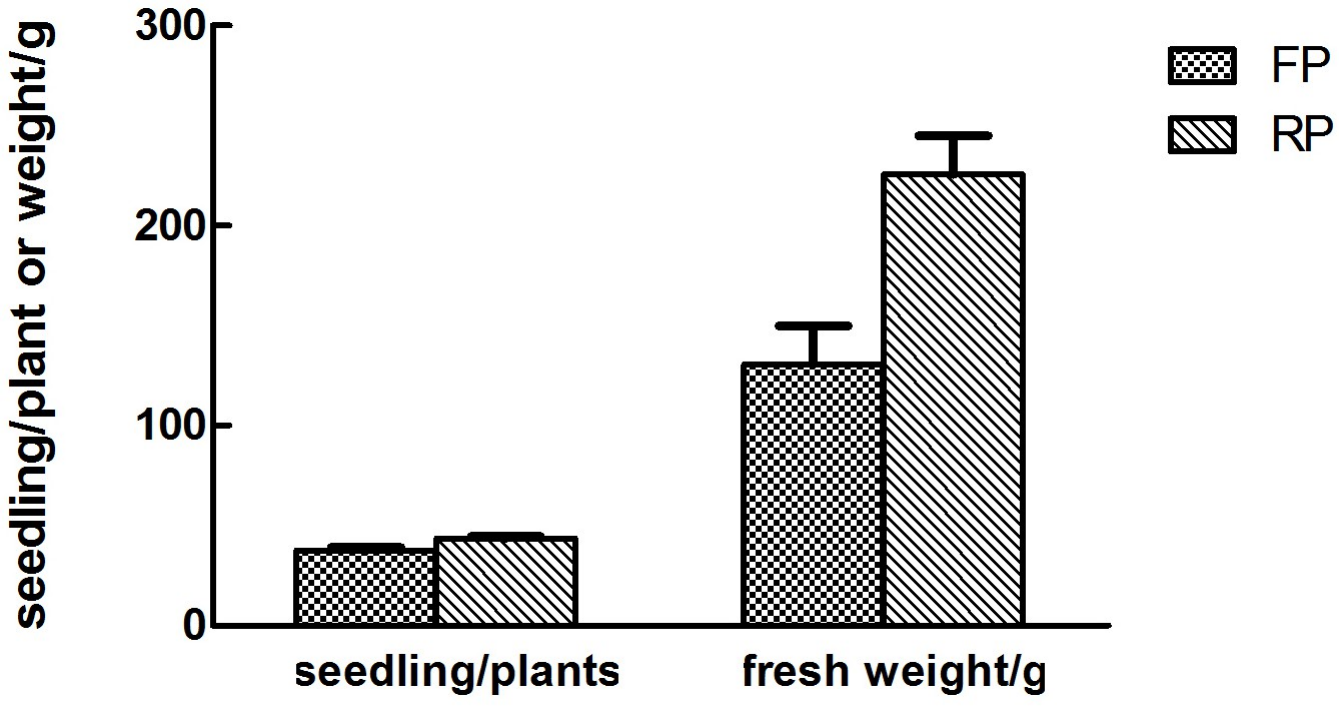
Different numbers of seedling and yield of *Pinellia ternata* with different planting patterns. RP is the abbreviation for ridge planting, and FP is the abbreviation for flat planting, same as below.

The number of *P*. *pinellia* seedlings before harvest in ridge planting was 43.73 plants /m row, significantly more than the number of seedlings of 37.73 plants /m row in flat planting (*t* = 3.6410, *p* = 0.0219). The yield of *P*. *pinellia* with flat planting was only 130.64 g/m row, while it was 225.60 g under ridge planting, which increased by 72.69% compared with flat planting (*t* = 3.5022, *p* = 0.0248) (Fig 1).

### Diversity of rhizosphere soil bacteria and fungi communities

A total of 395,703 bacterial 16S rRNA gene sequences and 477,579 fungal ITS sequences were obtained from 6 rhizosphere soil samples, with an average of 65,951 ± 1,047 bacterial sequences per soil sample and 79,597 ± 346 fungal sequences per soil sample. After deviations by different sequencing depths were removed, the resampled OTU tables were generated with 4053 bacterial 16S rRNA and 1852 fungal ITS reads. The shared and unique OTUs between the soils of ridge-planting and flat planting were significantly different in bacterial 16S rRNA, 802.00 ± 12.89 in flat planting and 549.00 ± 168.31 in ridge planting (*p* < 0.05), and no significant differences in fungal ITS OTUs (Table 1).

**Table 1:** The difference in OTUs and alpha diversity indices of bacterial 16S rRNA and fungal ITS between the soils of flat planting and ridge planting.

Microbial	Planting pattern	Alpha diversity indices				
		OTUs	Shannon	Simpson	Chao1	Coverage
bacteria	flat planting	802.00 a	8.96 a	1.00 a	821.62 a	1.00 a
	ridge planting	549.00 b	8.15 a	1.00 a	555.11 b	1.00 a
fungi	flat planting	306.33 a	3.96 a	0.76 a	315.09 a	1.00 a
	ridge planting	311.00 a	4.69 a	0.87 a	316.39 a	1.00 a

Note: Different lowercase letters within a column indicate significant differences among treatments according to *t*-test at 0.05 level.

The alpha diversity indices, including Shannon, Simpson, Chao1 were used to determine bacterial and fungal community diversity change. The Chao1 indices of the bacterial community in the flat planting soils were significantly higher than in ridge planting soil (*p* < 0.05). For the fungal community, no significant differences were detected between the flat-planting and ridge-planting soils (Table 1).

### Bacterial and fungal community structure analysis

**Fig 2:**
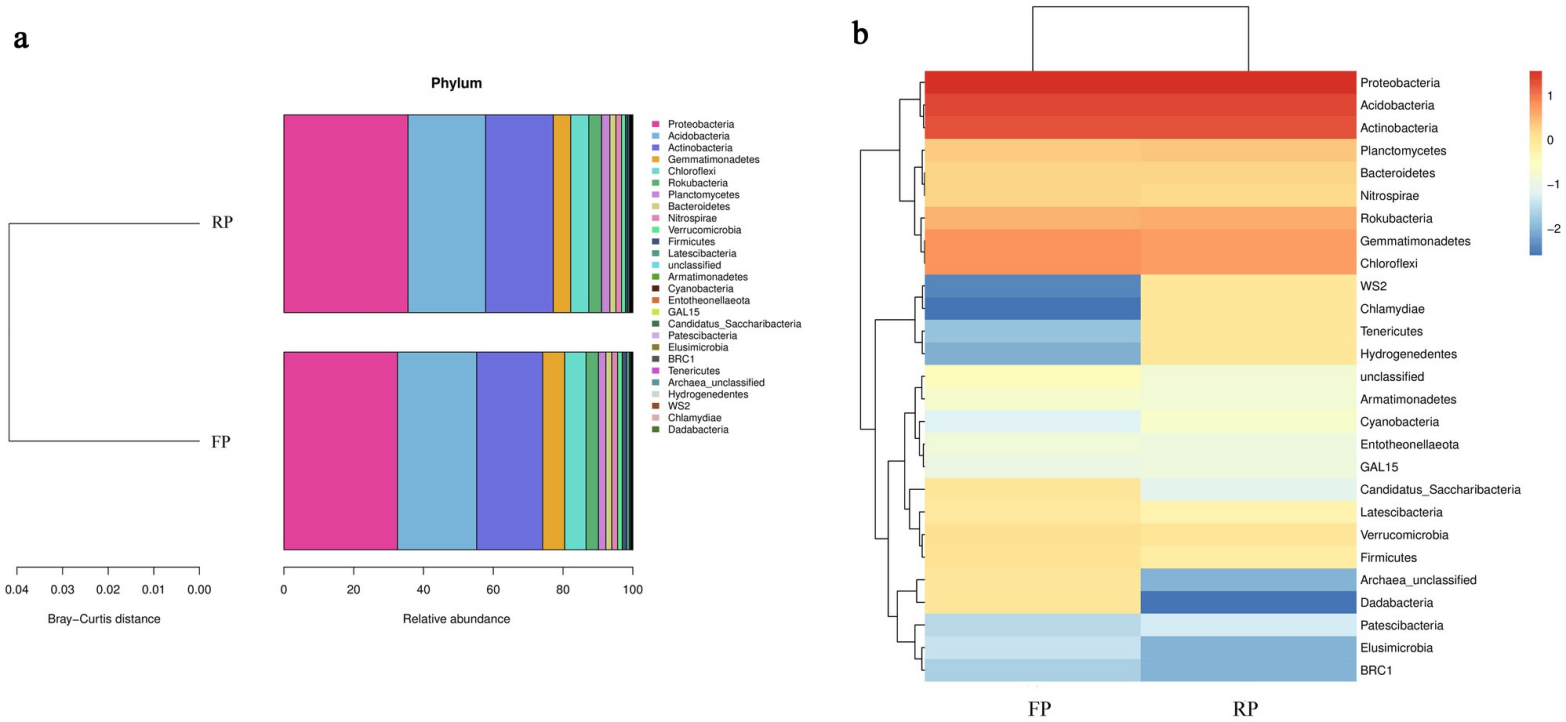
The 16S rRNA gene sequencing of the flat and ridge planting soils revealed bacterial community structure. (a) Relative abundance of phyla in the soils. Different colors indicate different phyla, and the abundance of each group is the average of all biological repeats within that group. (b) The detected bacteria at phyla level across all samples for Heat map analysis.

According to the OTU annotation results and OTU abundance tables, a phylogenetic tree for rhizospheric microorganisms (S1 Fig) and a relative abundance table of bacterial genes (Fig 2a) for phyla level were obtained. In the flat planting and ridge planting soils, 11 phyla had an abundance of more than 1%, *Proteobacteria* (32.55% and 35.57%), *Acidobacteria* (22.71% and 22.23%), *Actinobacteria* (18.91% and 19.38%) had the highest relative abundance. Compared to the highest 30 relative abundances bacterial at phyla level presented by the heat map, some bacteria like *Chlamydiae*, *Tenericutes*, and *Hydrogenedentes* were more abundant in ridge planting soil than in flat planting soil. However, there were still some opposite cases, such as *Dadabacteria* and *Archaea* (Fig 2b).

**Fig 3:**
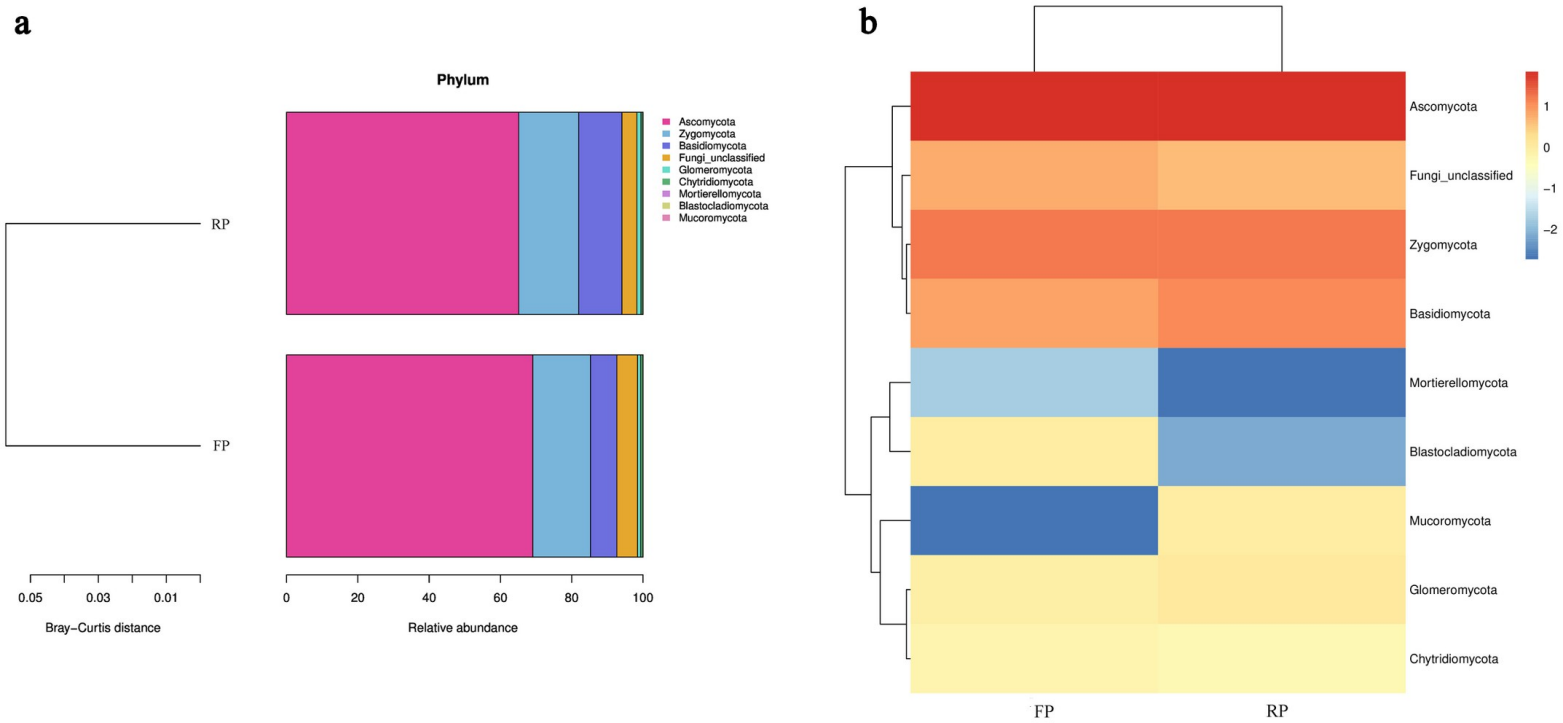
Fungal community structure revealed through ITS gene sequencing of the flat planting and ridge planting soils. (a) Relative abundance of phyla in the soils. Different colors indicate different phyla, and the abundance of each group is the average of all biological repeats within that group. (b) The detected fungi at phyla level across all samples for Heat map analysis.

The relative fungal abundance and the heat map analysis also showed similar appearances in the soils of flat and ridge planting. At the phyla level, nine phyla of eukaryotic microorganisms were annotated in the flat planting and ridge planting soil samples, of which *Ascomycota* (69.10% and 65.11%) was absolutely dominant, *Zygonycota* (16.17% and 16.85%) and *Basidiomycota* (7.40% and 12.15%) had more abundance (Fig 3a). *Zygomycota*, *Basidiomycota*, *Mucoromycota* and *Glomeromycota* had a higher relative abundance in the rhizosphere of ridge planting (Fig 3b).

### Analysis of rhizosphere microbial community composition

**Fig 4:**
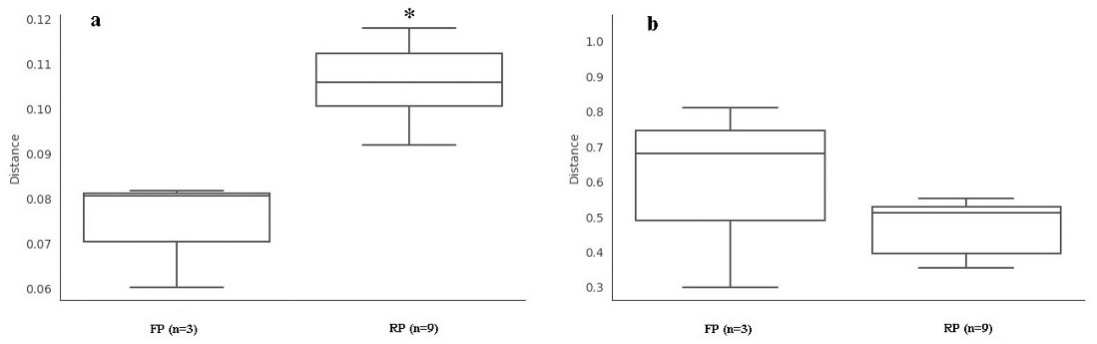
Box plot of the bacterial (a) and fungal (b) communities by adonis multivariate analysis of variance.

Adonis multivariate analysis of variance results of pairwise distances between bacterial (Fig 4a) and fungi (Fig 4b) communities, based on the bray-curtis distance, showed that the bacterial community in flat planting soil is significantly lower than that in ridge planting soil (*R*^*2*^ = 0.72, *P* < 0.05). The fungal community of flat planting shows no significant difference from ridge planting treatment (*R*^*2*^ = 0.26, *P* > 0.05). These suggested the flat planting and ridge planting soils’ bacterial community composition varied significantly.

**Fig 5:**
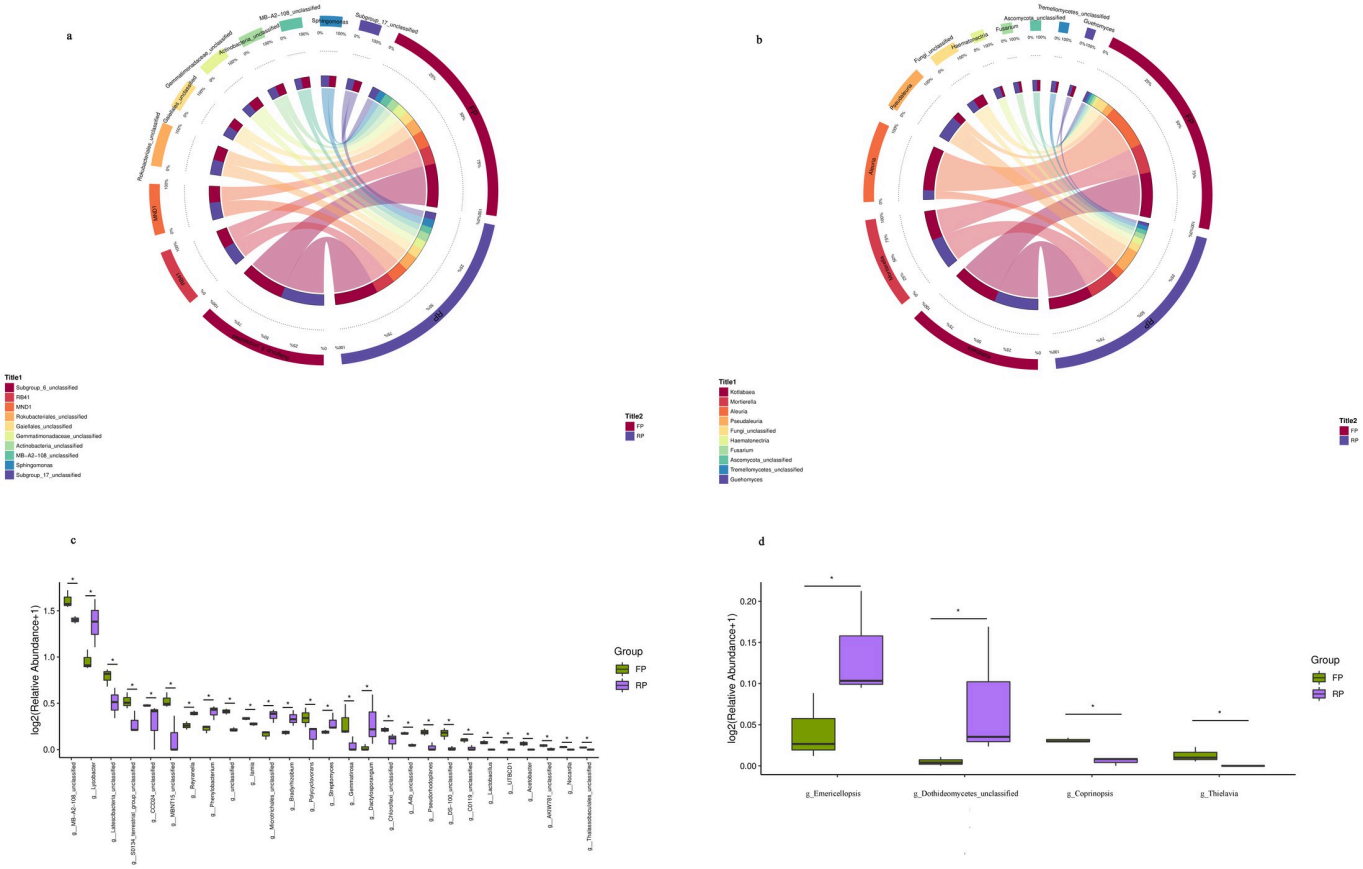
Relative abundance of rhizosphere microbialgenus under flat planting and ridge planting treatments. Chord diagram showing the interrelationships between relative abundance of top 10 bacterial (a) and fungal (b) genera under different treatments. The change in the width of the color bands indicates the change in the relative abundance of bacterial and fungal communities. Bar plots show the significant differences in the relative abundance of bacterial (c) and fungal (d) genera under different treatments. Asterisks indicates a significant difference between the two treatments.

The rhizosphere community composition significantly changed at the genus level under different cropping systems. A chord diagram showed the interrelationship between the relative abundance of the 10 most dominant bacterial (Fig 5a) and fungal (Fig 5b) communities at the genera level under flat and ridge planting. *Subgroup_6*, *RB41*, and *MND1* were the most dominant bacterial genera in the rhizosphere of flat and ridge planting (Fig 5a). *Kotlabaea* and *Mortierella* were the dominant fungal genera in both treatments, and *Aleuria* was more abundant in flat planting (Fig 5b). Significant differences in the relative abundance of bacterial (Fig 5c) and fungal (Fig 5d) communities at the genus level are represented by bar plots (Wilcoxon test, *P* < 0.05). Compared with the bacterial community in flat planting soil, 7 bacterial genera were up-regulated, and 22 were down-regulated in ridge planting soil (Fig 5c). Seven up-regulated bacterial genera in descending order were *Lysobacter*, *Reyranella*, *Phenylobacterium*, *Microtrichales*, *Bradyrhizobium*, *Streptomyces*, and *Dactylosporangium* (Wilcoxon test, *P* < 0.05). Five bacterial genera were only found in the flat planting soil, including the genus of *Lactobacillus*, *UTBCD1*, *Acetobacter*, *Nocardia*, and *Thalassobaculales* (Wilcoxon test, *P* < 0.05). About the fungal community, only 4 fungal genera were significantly up/down-regulated in the two soil treatments. In the four fungal genera, *Emericellopsis* and a fungal genus from Dothideomycetes were significantly up-regulated in the ridge planting soil. Still, *Thielavia* and *Coprinopsis* did not determine ridge planting soil (Wilcoxon test, *P* < 0.05, Fig 5d).

### Effect of ridge planting on the BCAs

**Fig 6:**
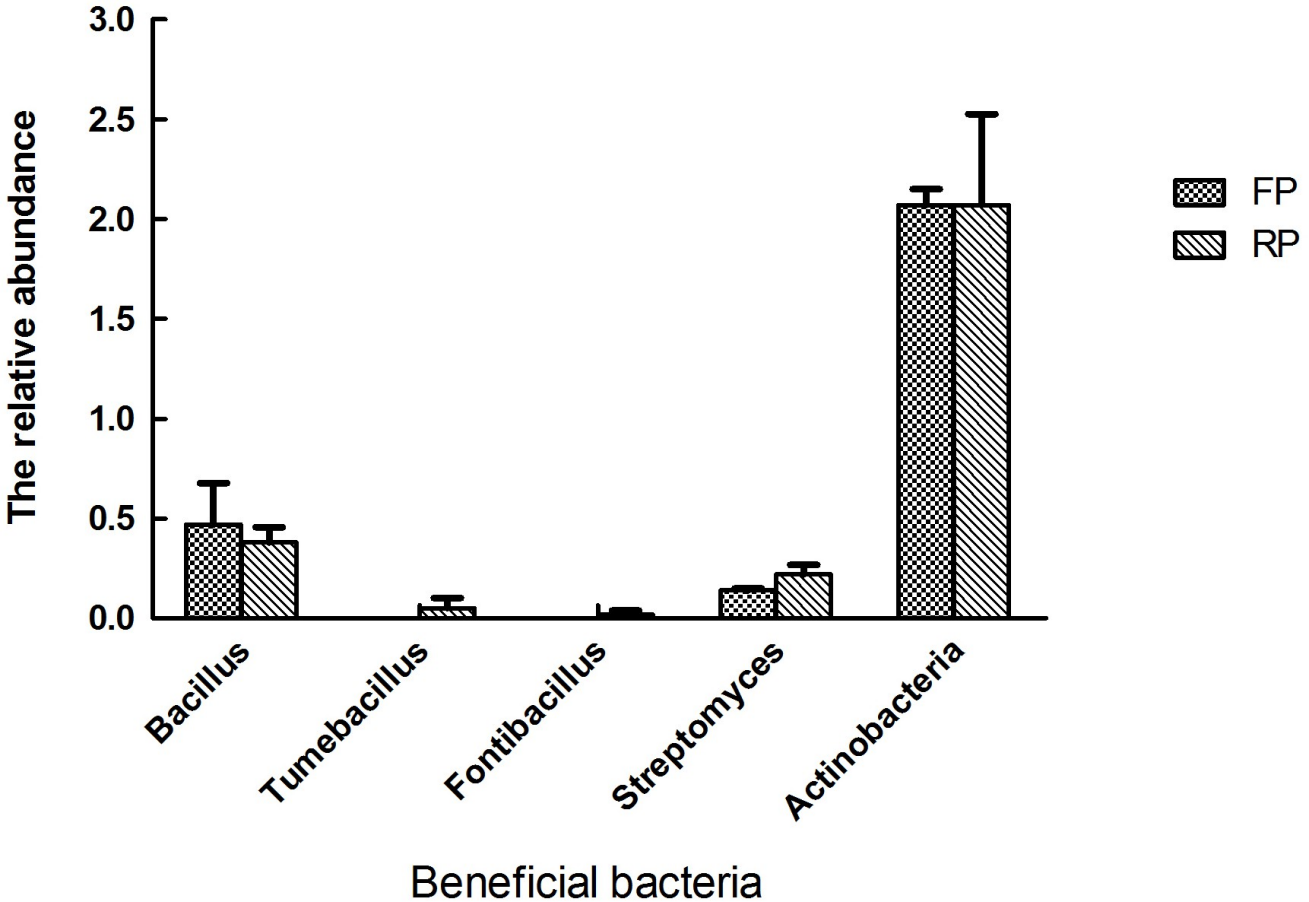
The relative abundance of several biocontrol agents in the flat planting and ridge planting rhizosphere soils.

Some soil microbes serve as biocontrol agents (BCAs). They include several bacterial genera, such as *Bacillus*, *Tumebacillus*, *Fontibacillus*, *Actinobacteria* and *Streptomyces* [39, 40]. In the five BCAs, *Streptomyces* is significantly higher in ridge planting soil than flat planting soil (Wilcoxon test, *P* < 0.05, Fig 6). *Tumebacillus* and *Fontibacillus* are all up-regulated in ridge planting soil. *Bacillus* and *Actinobacteria* have no obvious change in two soils, even though *Bacillus* is down-regulated in ridge planting soil.

### Relationship between rhizosphere microbiome and yield

**Fig 7:**
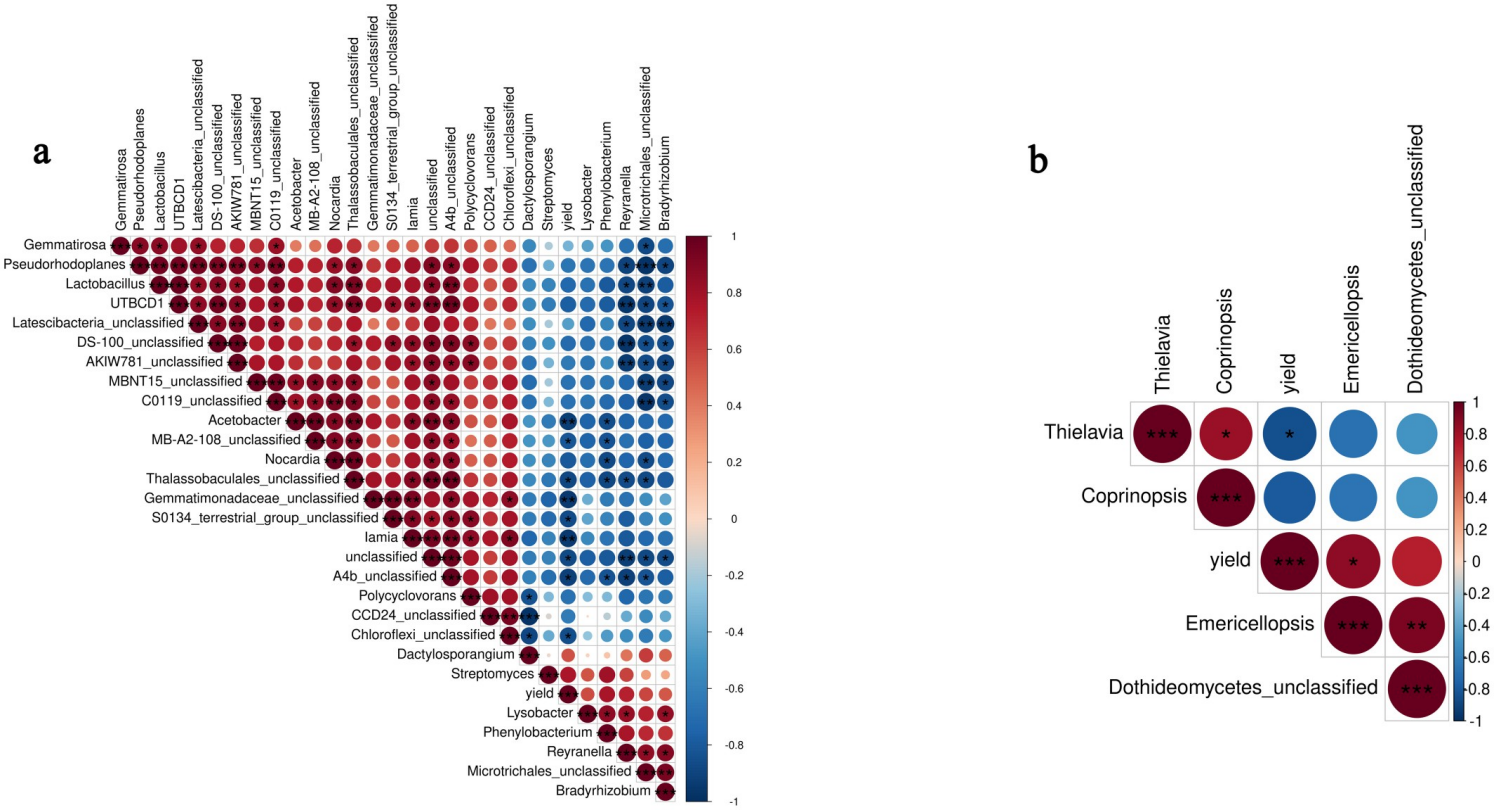
Correlation analysis between rhizophere microbime and yield according to Pearson correlation coefficient (PCC, *P* < 0.05). PCC between bacterial genera and yield (a), and PCC between fungal genera and yield (b). Asterisks indicates significant differences (*, *P* < 0.05; **, *P* < 0.01; ***, *P* < 0.001).

Based on the results of rhizosphere soil microbial composition and relative abundance, to further explore the influence of these microorganisms on pinellia yield, a correlation analysis was performed at the genera level according to the Pearson correlation coefficient (PCC, Fig 7). The PCC results revealed that bacterial genera *Acetobacter*, *Iamia*, and a bacterial genus from Gemmatimonadaceae (*P* < 0.01, Fig 7a) and fungal genera *Thielavia* (*P* < 0.05, Fig 7b) were negatively correlated with the yield. Moreover, *Acetobacter* and *Thielavia* only found in rhizosphere soil of flat planting. This suggested that the microbiome may provide an unfriendly environment for plant growth. In contrast, the fungal genera *Emericellopsis* was positively correlated (*P* < 0.05, Fig 7b) with yield, indicating that *Emericellopsis* was beneficial to the growth of *P*. *ternate*. Meanwhile, it was found more abundance in ridge planting soil.

## Discussion

The current study showed that ridge planting can increase the yield of *P*. *ternata*. Therefore, we hypothesized that the physical environment of the soil changed after ridging, making it more suitable for root growth, especially in the rainy season. In the experimental area, 49.16% of rainfall appeared in August during the growth period from April to October (S1 Table). Since rainfall is the main characteristic of the growing season in the agricultural growing areas of North China, the main concern for local farmers is how to drain the water during the rainy season to prevent their crops from submerging for a long time. The purpose of this planting pattern differs from the use of ridge film mulching and furrow planting. Its goal is to effectively utilize and concentrate rainwater in dry semi-humid areas during the rainy season [15, 41].

The rhizosphere microbiome is a key factor in plant health and microenvironment stability, and the diversity and community composition vary with the changes in the soil environment [42, 43]. The soil bacterial community, as the most abundant microbial community in the soil, plays a crucial role in the occurrence of crop diseases. Healthy soils exhibited higher microbial diversity, 22 phyla including *Proteobacteria*, *Actinobacteria*, *Chloroflexi*, *Firmicutes* were more abundant in the healthy soils than the bacterial wilt infected soils [44]. The decrease of bacterial diversity and significant changes in some keystone taxa abundances such as *Acidobacteria*, *Phenylobacterium* and *Solirubrobacter*, may be important factors leading to increased soil-borne diseases and decreased production potential or quality of tobacco under continuous cropping pattern [27]. In this study, the soil bacterial community composition in the ridge planting differed greatly from flat planting. In contrast, the changes in the fungal community were not significant. In general, 29 bacteria communities varied significantly in ridge planting soil. *Dactylosporangium*, *Microtrichales*, *Bradyrhizobium*, *Phenylobacterium*, *Lysobacter*, *Streptomyces*, and *Reyranella* are the main up-regulated bacteria genera. *DS-100*, *Pseudorhodoplanes*, *Gemmatirosa*, and 19 other bacteria are the main down-regulated bacteria genera. Further, correlation analysis showed that bacterial and fungal genera, including *Acetobacter*, *Iamia* and *Thielavia*, as well as some unknown bacteria from Chloroflexi, A4b, MB-A2-108 and so on, were negatively correlated (P < 0.05) with pinellia yield. These microbiomes may increase the population of pathogens or provide an unfavorable environment for plant growth, leading to a significant reduction in production [27, 45]. The interaction between *Acetobacter*, *Iamia* and *Thielavia* related to yield reduction is unclear and warrants further study. Fortunately, we also found *Emericellopsis*, a genus of Ascomycota fungi within the order Hypocreales [46], was positively associated with production of *P*. *ternate*. Some studies showed that the new natural antibacterial and antifungal compounds were isolated from *Emericellopsis* against pathogens resistant to multiple agents [46, 47]. These biologically active components or *Emericellopsis* in the rhizosphere soil may benefit the plants, affecting their ability to cope with stress and thus promoting plant growth. Some soil microbes are beneficial to plants and protect the plant from soil-borne pathogens by producing antibiotics to inhibit the reproduction and development of pathogenic fungi. *Bacillus*, *Tumebacillus*, *Fontibacillus*, *Actinobacteria*, and *Streptomyces* are the main biocontrol agents (BCAs) [39, 40, 44]. In our study, *Streptomyces* is significantly up-regulated in ridge planting soil, while the others were not significantly different in the two soils. The genus *Streptomyces* is the most important source of bioactive natural products for pharmaceutical and agricultural applications [48, 49], accounting for 39% of all the reported microbial metabolites [50]. These BCAs could act as key taxa, reducing the chance of plant soil-borne pathogen invasion and reshaping the structure and composition of the rhizosphere microbiome [51, 52].

Based on this research, it was shown that the cultivation pattern of ridge planting can significantly regulate the changes in microbial community composition in soil. It makes the soil microenvironment more conducive to the growth of crop roots. Ridge planting can also improve the soil structure of the plough layer and the farmland microclimate, raise soil temperature, promote the development of roots, and increase dry matter accumulation [14]. However, this cultivation pattern is more suitable for areas with irrigated conditions and high concentrations of monsoon rainfall. Whereas under rain-fed conditions in dry semi-humid areas, tie ridges and furrow planting is an effective planting strategy for sustainable agriculture. The tied ridges conserve more rainwater during the rainy season and increase soil moisture content, supporting plant growth [15, 53]. Therefore, crop growers must choose the appropriate planting mode according to the local agricultural conditions and climate characteristics.

## Conclusions

In summary, we concluded ridge planting can effectively avoid the negative effects of soil moisture on *P*. *ternate* during the rainy season. Increase the number of seedlings and improve the yield compared with flat planting. Ridge planting can change the diversity and composition of rhizosphere bacterial microbial communities by increasing the α-diversity indices and the relative abundance of bacterial consortia, such as *Chlamydiae*, *Tenericutes*, and *Hydrogenedentes*. No significant differences in fungal community were detected between the flat and ridge planting soils. In addition, biocontrol agents *Streptomyces* are significantly higher in ridge planting soil than in flat planting soil. Correlation analysis revealed the yield of *P*. *ternata* was positively correlated with fungal genera *Emericellopsis*, which had more abundance in ridge planting, and negatively correlated with bacterial genera *Acetobacter*, *Iamia*, and fungal genera *Thielavia*. *Acetobacter* and *Thielavia* can only be found in flat planting. Therefore, ridge planting was more beneficial to *P*. *ternate* growth by changing the diversity and composition of rhizosphere microbiome. This study is of great significance for formulating targeted management measures to mitigate or overcome the damage caused by excessive humidity in the rainy season to *P*. *ternate* and providing a theoretical basis for screening rhizosphere functional strains.

## Supporting information

S1 Table Monthly mean temperature and rainfall from April to October 2020 in Baoding. (DOCX)

S1 Fig Phylogentic tree for rhizospheric microorganisms at the phylum level. (a) bacteria, (b) fungi.
(TIF)
