# Supplementary material for: Ridge planting increases the rhizosphere microbiome diversity and improves the yield of Pinellia ternata (Thunb.) Breit in North China
Source: PLoS One. 2024 Sep 13;19(9):e0304898. doi: 10.1371/journal.pone.0304898 (PMC11398693; doi:10.1371/journal.pone.0304898)
Supplement: S1 Table — (DOCX) [file pone.0304898.s001.docx]

| month | mean temperature/℃ | rainfall /mm |
| --- | --- | --- |
| Apr-20 | 15.2 | 32.0 |
| May-20 | 20.8 | 53.1 |
| Jun-20 | 26.5 | 2.4 |
| Jul-20 | 25.4 | 32.1 |
| Aug-20 | 25.2 | 170.2 |
| Sep-20 | 20.4 | 56.2 |
| Oct-20 | 12.0 | 0.2 |

Table S1 Monthly mean temperature and rainfall from April to October 2020 in Baoding
