# Supplementary figures and images for: Ridge planting increases the rhizosphere microbiome diversity and improves the yield of Pinellia ternata (Thunb.) Breit in North China
Source: PLoS One. 2024 Sep 13;19(9):e0304898. doi: 10.1371/journal.pone.0304898 (PMC11398693; doi:10.1371/journal.pone.0304898)

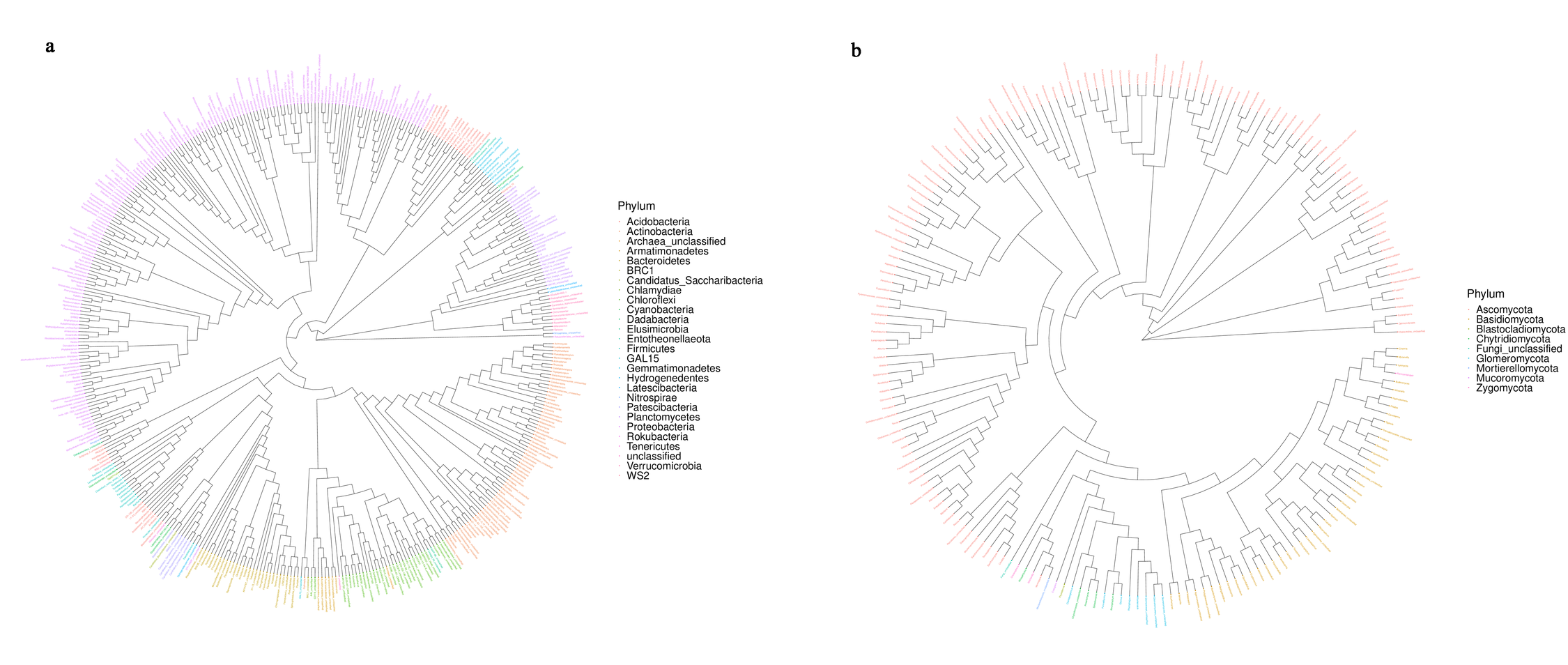

Supplement: S1 Fig — (a) bacteria, (b) fungi. (TIF) [file pone.0304898.s002.tif]
